# Supplementary material for: Superinfection exclusion: A viral strategy with short-term benefits and long-term drawbacks
Source: PLoS Comput Biol. 2022 May 10;18(5):e1010125. doi: 10.1371/journal.pcbi.1010125 (PMC9122224; doi:10.1371/journal.pcbi.1010125)
Supplement: S1 Appendix — Here we discuss the decision to not incorporate stochasticity in lysis time in the model presented in the main text. (PDF) [file pcbi.1010125.s005.pdf]

Stochasticity in Lysis Time

In the main text we choose to implement stochasticity in both adsorption and burst size, but not in lysis time. The reason we make this choice is that to introduce stochasticity in lysis time in a realistic way would require additional parameters. For instance, Campos *et al.* compare descriptions of lysis using piece-wise, Gaussian and logistic-like functions [1]. Depending on the mathematical framing of these functions, each would require at least one parameter that controls the width or rise rate of the distribution. Given that our model already contains several parameters, we preferred to choose a simpler description, where lysis time is deterministic.

Nevertheless, here we implement a version of the model described in the main text where we introduce stochasticity in lysis time. In this version of the model, at the point of infection, a lysis time L is drawn from a Gaussian distribution with mean τ and standard deviation $\tau/10$. The choice of standard deviation is based on lysis time data collected for different variants of coliphage T7 (Fig A1). In this case the variants are an isolate of wild-type T7 originally obtained as an aliquot from the Richardson Lab (Harvard Medical School, Boston, MA), and T7 mutant D111 (wild-type T7 background with deletions from base 532 to 1662) which was kindly donated by Dr. Vivek Mutalik from the Berkeley Lab (Berkeley, CA). Briefly, the technique used here is adapted from the single-step growth curve protocol used originally by Ellis and Delbrück [2], and involves infecting an exponentially growing culture of bacteria with phage and then periodically sampling, with the samples being plated in soft agar with susceptible host to attain plaques. Counting the number of plaques yields the plaque forming units (PFUs) as a function of time, and from this the lysis time and burst size can be inferred.

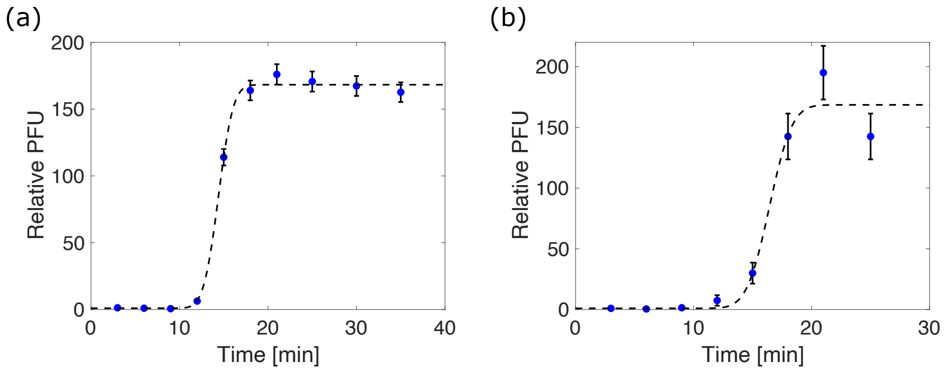

Figure A1: One-step growth curves of two coliphage T7 variants. (a) corresponds to an isolate of wild-type T7, while (b) corresponds to T7 mutant D111 which is the wild-type T7 background with deletions from base 532 to 1662. Fit to the data is a Gaussian function, with mean lysis time τ, standard deviation σ and burst size β. These fits yield $\tau_a = 14.40(11)$ min, $\sigma_a = 1.32(19)$, $\beta_a = 168(2)$, $\tau_b = 16.4(5)$ min, $\sigma_b = 1.5(5)$ and $\beta_b = 168(12)$. For this data it can therefore be seen that $\sigma \sim \tau/10$.

Using this version of the model, we re-run a subset of our simulations in the main text, namely the simulations used to determine the probability of fixation of neutral mutants. As can be seen in Figs A2 and A3, the introduction of stochasticity in lysis time does not significantly alter the behaviour of the model. It is still the case that the probability of fixation of a neutral mutant is controlled by its initial frequency in the population (Fig A2), and that the average behaviour of the model remains unchanged, with the simulations remaining consistent with an ODE description (S2 Appendix) of the model (Fig A3). We do note that the difference between superinfecting and superinfection excluding scenarios in Fig A2 is less clear than in the corresponding figure in the main text. We speculate that this is caused by the increased level of stochasticity introduced here, but a more detailed analysis would be required to test this systematically.

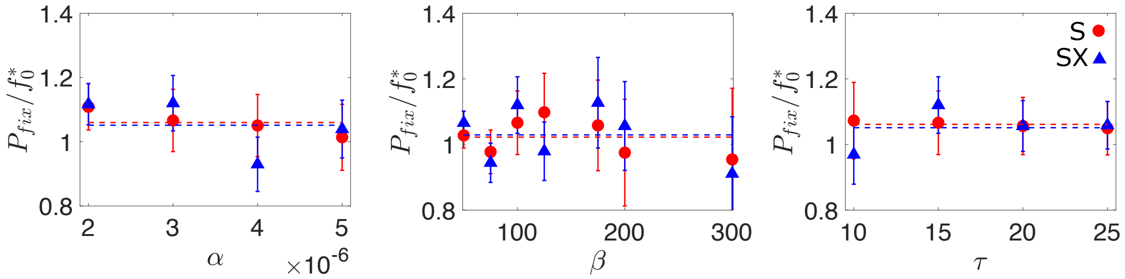

Figure A2: Probability of mutant fixation P_{fix} in the superinfecting (S) and non superinfection exclusing (SX) scenarios, scaled by the initial frequency of the mutant $f_0^* = 1/(V_{ss} + \beta I_{ss})$, as a function of adsorption rate α, burst size β and lysis time τ. Dashed lines indicate the simple average of the data for both the superinfecting (blue) and superinfection-excluding (red) scenarios. Unless otherwise stated, the parameters used were $\alpha = 3 \times 10^{-6}$, $\beta = 100$, $\tau = 15$, $\delta = 0.1$ and $B_0 = 1000$. The data is obtained from a minimum of 10 million independent simulations.

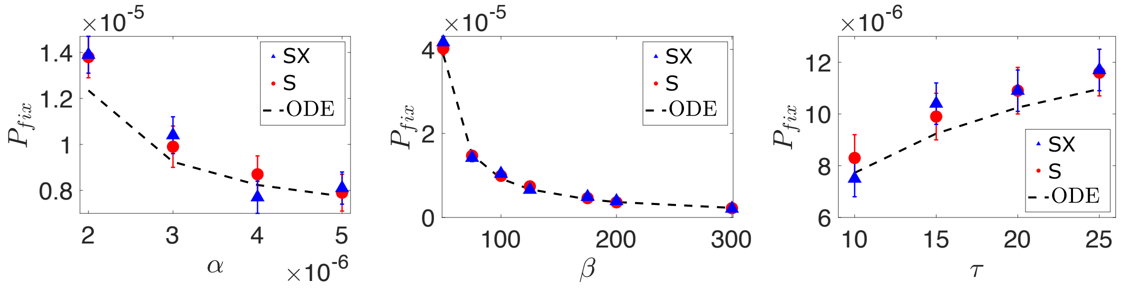

Figure A3: Probability of mutant fixation P_{fix} in the superinfection (S) and superinfection-exclusion (SX) scenarios as a function of adsorption rate α, burst size β and lysis time τ. Error bars are plotted, although in some instances may be too small to see. This data is compared with the solution of a system of ODEs used to describe the average behaviour of the model (Appendix 2), where the black dashed line represents the frequency f_0^* calculated from the steady-state values. Unless otherwise stated, the parameters used were $\alpha = 3 \times 10^{-6}$, $\beta = 100$, $\tau = 15$, $\delta = 0.1$ and $B_0 = 1000$. The data is obtained from a minimum of 10 million independent simulations.

References

[1] Campos D, Méndez V, Fedotov S. The effects of distributed life cycles on the dynamics of viral infections. Journal of Theoretical Biology. 2008;254(2). doi:10.1016/J.JTBI.2008.05.035.

[2] Ellis EL, Delbrück M The Growth of Bacteriophage. The Journal of general physiology. 1939;22(3). doi:10.1085/JGP.22.3.365.
