# Supplementary material for: Superinfection exclusion: A viral strategy with short-term benefits and long-term drawbacks
Source: PLoS Comput Biol. 2022 May 10;18(5):e1010125. doi: 10.1371/journal.pcbi.1010125 (PMC9122224; doi:10.1371/journal.pcbi.1010125)
Supplement: S2 Appendix — The average behaviour of the model used in the main text is described by a set of ordinary differential equations (ODEs), showing good agreement with our stochastic simulations. (PDF) [file pcbi.1010125.s006.pdf]

ODE Description of Model

The average behaviour of the model used in the main text can be described by a set of ordinary differential equations (ODEs):

$$\frac{dV}{dt} = -\alpha V(B+I) - \delta V + \beta\alpha V_{t-\tau}B_{t-\tau}, \tag{1a}$$

$$\frac{dB}{dt} = -\alpha VB + \alpha V_{t-\tau}B_{t-\tau}, \tag{1b}$$

$$\frac{dI}{dt} = \alpha VB - \alpha V_{t-\tau}B_{t-\tau}, \tag{1c}$$

where all of the symbols are defined the same as in the main text (V, B and I indicate the concentrations of phage, uninfected bacteria and infected bacteria as a function of time respectively; α, β, τ and δ indicate the phage adsorption rate, burst size, lysis time and decay rate respectively). The subscript is used to indicate that those terms are calculated at time $t-\tau$. The positive term in Eq. 1b accounts for the instantaneous replacement of lysed cells in our turbidostat environment.

By numerically solving this ODE system, we can verify that for the parameters used in this work, a steady state solution is reached where $V = V_{ss}$, $B = B_{ss}$ and $I = I_{ss}$, in agreement with the average behaviour of the stochastic model used throughout the main text (Figs B1 and B2).

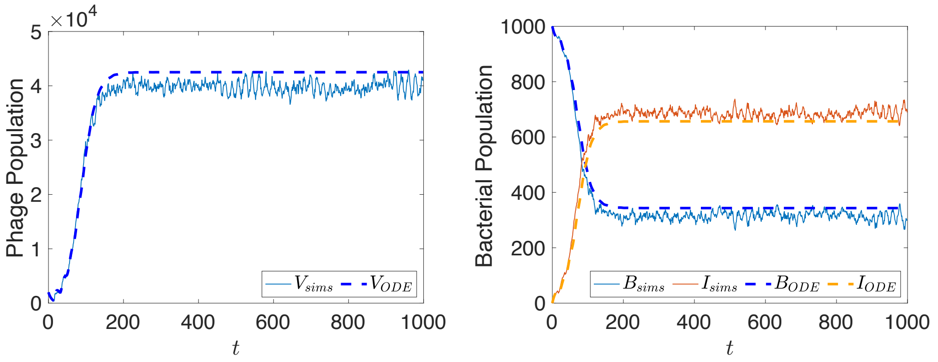

Figure B1: The average behaviour of the model in the main text is mostly captured by the ODE description set out in Eqs. 1. Slight discrepancies probably arise from the discreteness of the infection, decay and lysis steps in the simulations. Parameters used are $\alpha = 3 \times 10^{-6}$, $\beta = 100$, $\tau = 15$, $\delta = 0.1$ and $B_0 = 1000$.

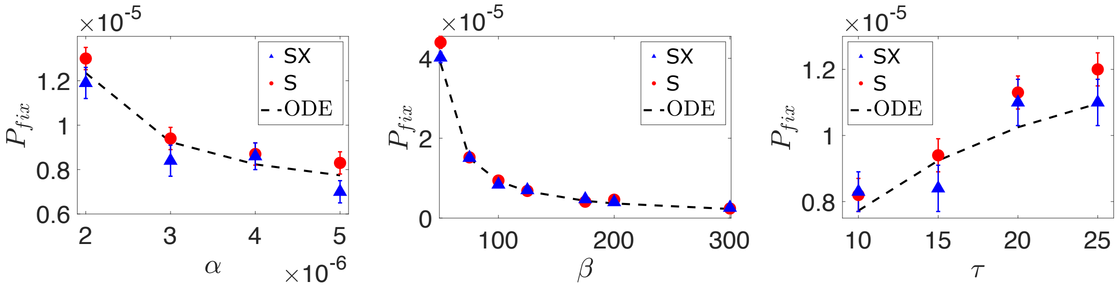

Figure B2: Probability of mutant fixation P_{fix} in the superinfection (S) and superinfection-exclusion (SX) scenarios as a function of adsorption rate α, burst size β and lysis time τ. This is the same as the data displayed in Fig 3 of the main text, prior to rescaling by the initial frequency $f_0^* = 1/(V_{ss} + \beta I_{ss})$. Error bars are plotted, although in some instances may be too small to see. This data is compared with the solution of the system of ODEs, where the black dashed line represents the frequency f_0^* calculated from the steady-state values.
