# Supplementary material for: Superinfection exclusion: A viral strategy with short-term benefits and long-term drawbacks
Source: PLoS Comput Biol. 2022 May 10;18(5):e1010125. doi: 10.1371/journal.pcbi.1010125 (PMC9122224; doi:10.1371/journal.pcbi.1010125)
Supplement: S3 Appendix — A quantitative comparison between the fixation probabilities obtained in our stochastic simulations with those that would be predicted in a similarly parameterised Moran model. (PDF) [file pcbi.1010125.s007.pdf]

Comparison To Moran Model

If we introduce a free scaling parameter ϕ, such that $s_{comp} = \phi s_{growth}$, and optimally fit Eq. 1 in the main text to the data, we can compare the resulting values for ϕ with our previous estimates of σ to measure of the quality of agreement between simulations and theory. It should be noted that while we have described the scaling in terms of s_{comp}, it is mathematically identical to scaling N_e. Through this optimal fitting we find that $\phi_{S\alpha} = 1.2226$, $\phi_{SX\alpha} = 1.3406$, $\phi_{S\beta} = 1.0321$, $\phi_{SX\beta} = 0.9391$, $\phi_{S\tau} = 0.2681$ and $\phi_{SX\tau} = 0.0076$ with subscripts indicating scenario and parameter combinations. This indicates that the data maps well to an equivalently parameterised Moran model, with an average difference of $\sim 5\%$.
