# Supplementary material for: Superinfection exclusion: A viral strategy with short-term benefits and long-term drawbacks
Source: PLoS Comput Biol. 2022 May 10;18(5):e1010125. doi: 10.1371/journal.pcbi.1010125 (PMC9122224; doi:10.1371/journal.pcbi.1010125)
Supplement: S4 Appendix — Here we repeat a subset of the measurements carried out in the main text with different resident phage parameters, in this instance βres = 70. (PDF) [file pcbi.1010125.s008.pdf]

$\beta_{res} = 70$ Measurements

Here we repeat a subset of the measurements carried out in the main text for different resident phage parameters, in this instance $\beta_{res} = 70$, with all other parameters remaining the same as in the main text. First, the effective population size is measured in both superinfecting and superinfection-excluding populations (Fig D1), demonstrating that N_e is larger in superinfecting populations.

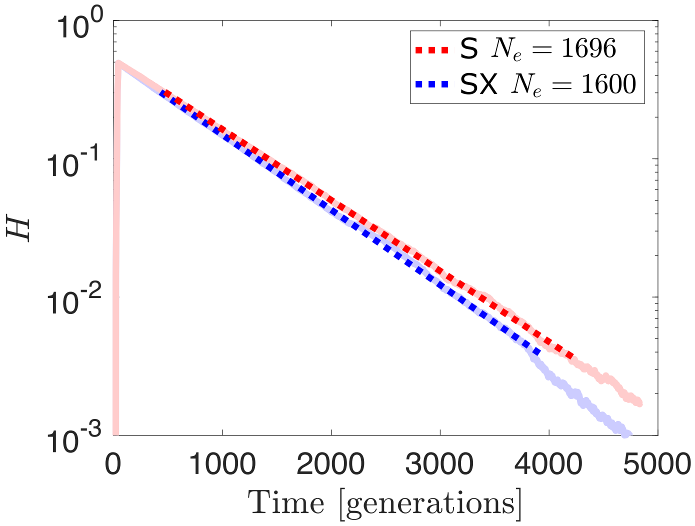

Figure D1: Linear fit to log transformed heterozygosity data, with slope $\Lambda \equiv 2/N_e$ revealing that allowing superinfection (red) results in a larger effective population size compared to the case where superinfection is prevented (blue). Parameters used were $\alpha = 3 \times 10^{-6}$, $\beta = 70$, $\tau = 15$, $\delta = 0.1$ and $B_0 = 1000$.

We then move on to characterise the fitness of non-neutral mutants, in this instance only varying burst size β (Fig D2). Again, we find a positive linear relationship between burst size and fitness, both in terms of the effect on growth rate in isolation and in a competitive setting. Interestingly here we find that alterations to burst size make slightly less difference in a competitive setting, as compared to the effect on growth rate. This could potentially be because, at lower burst sizes, any small change in β has a large impact on the growth rate, but has a smaller impact in a population already at steady state.

Finally, we put both aspects together and measure the probability of fixation of non-neutral mutants in both superinfecting and superinfection-excluding populations (Fig D3). As in the main text, we find fairly good, although slightly worse, agreement between our simulation results and the prediction from a Moran model with our independently measured parameters (Figs D1 and D2). In terms of the additional fitting parameter introduced in S3 Appendix, we find here that $\phi_S = 0.8021$ and $\phi_{SX} = 0.7269$. It's possible that this discrepancy is caused by imprecision in the measurements of fitness as a function of burst size. Indeed, over the whole range of β we would not expect a perfect linear relationship between burst size and fitness, with the benefits of increased burst size being larger for small β, and so at these lower values of β we find that the linear fit is less of a good approximation.

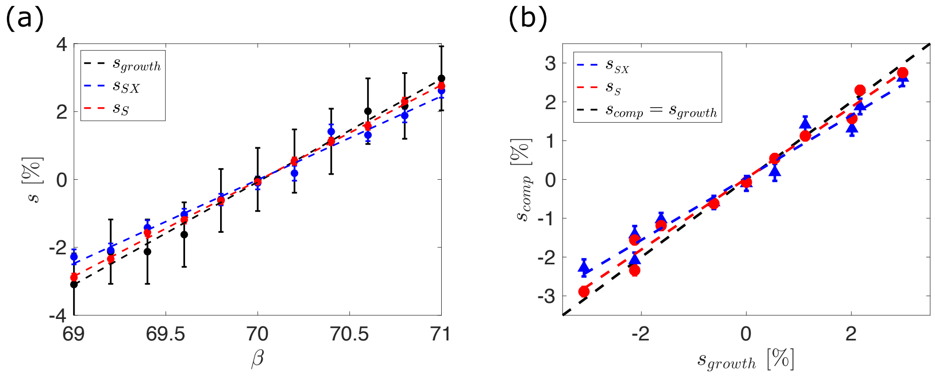

Figure D2: (a): The selective advantage s relative to a resident phage that results from a change to burst size β. This is measured both in terms of the effect on the isolated growth rate of the mutant (s_{growth}, Eq. 8 in main text), and in terms of the change in frequency in a population initiated with 50% mutant and 50% resident (s_{SX} and s_S, Eq. 9 in main text). (b): The fitness in a competitive setting s_{comp} is then shown as a function of the fitness in an isolated setting s_{growth}. Straight line fits are shown as dashed lines, with gradient σ such that $s_{comp} = \sigma s_{growth}$. From the above data we find $\sigma_S = 0.9181$ and $\sigma_{SX} = 0.8032$. Resident parameters used were $\alpha = 3 \times 10^{-6}$, $\beta = 70$ and $\tau = 15$. As before $\delta = 0.1$ and $B_0 = 1000$. s_{growth} determined from 500 simulations, and s_{comp} determined from 200 simulations. Error bars are given by the standard error on the mean of the simulations.

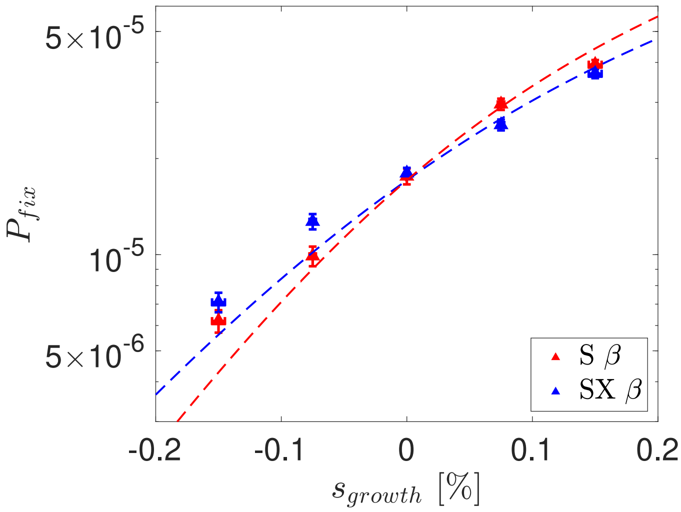

Figure D3: Probability of mutant fixation P_{fix} as a function of selective growth advantage s_{growth}. Points indicate simulation results, while lines indicate theoretically predicted values in a Moran model with equivalent parameters (Eq. 1 in main text). The error in our estimate of the fixation probability ΔP_{fix} is given by $\Delta P_{fix} = \sqrt{n_{fix}}/n$, where n and n_{fix} represent the total number of simulations and the number of simulations where the mutant fixes respectively. Error bars in the x-axis represent the errors on the growth rate fitness s_{growth} that each burst size corresponds to. These are calculated by fitting a linear relation to growth rate measurements such that $s_{growth} = m(\beta_{mut} - \beta_{res})$. The fractional error on the s_{growth} is then equal to the fractional error on the fitted gradient m. The data is obtained from a minimum of 20 million independent simulations.
