# Supplementary material for: Superinfection exclusion: A viral strategy with short-term benefits and long-term drawbacks
Source: PLoS Comput Biol. 2022 May 10;18(5):e1010125. doi: 10.1371/journal.pcbi.1010125 (PMC9122224; doi:10.1371/journal.pcbi.1010125)
Supplement: S5 Appendix — Here we support the generation time calculated in the main text with results of stochastic simulations. We also include a more detailed discussion about the differences in generation time between superinfecting and superinfection-excluding populations. (PDF) [file pcbi.1010125.s009.pdf]

Calculation of Generation Time

In the main text we show that the generation time T can be written as

$$T = \tau + \frac{1}{\delta + \alpha I_{ss}}. \tag{1}$$

This yields $T = 24.80$ for the parameters used in the main text ($\alpha = 3 \times 10^{-6}$, $\tau = 15$, $\delta = 0.1$ and $I_{ss} = 681$).

We here verify the results of this expression using stochastic simulations. We simulate a single phage, which in each time-step Δt has a probability of successfully adsorbing to an uninfected host ($\alpha B_{ss} \Delta t$), and a probability of *dying* ($(\delta + \alpha I_{ss})\Delta t$). In the event that the phage successfully adsorbs to a host before it dies, the number of steps t_{steps} taken for this to occur is noted, and the time $T = \tau + t_{steps}$ is recorded (representing the time between the 'birth' of the original phage and the 'birth' of it's offspring, as per the definition of generation time in the main text). A schematic representation of this process is shown in Fig E1. This process was repeated 10 million times, with the the time T being recorded in all of the instances where the phage successfully reproduced. This yields an average generation time of $\overline{T} = 24.78(3)$ in agreement with the analytical calculation.

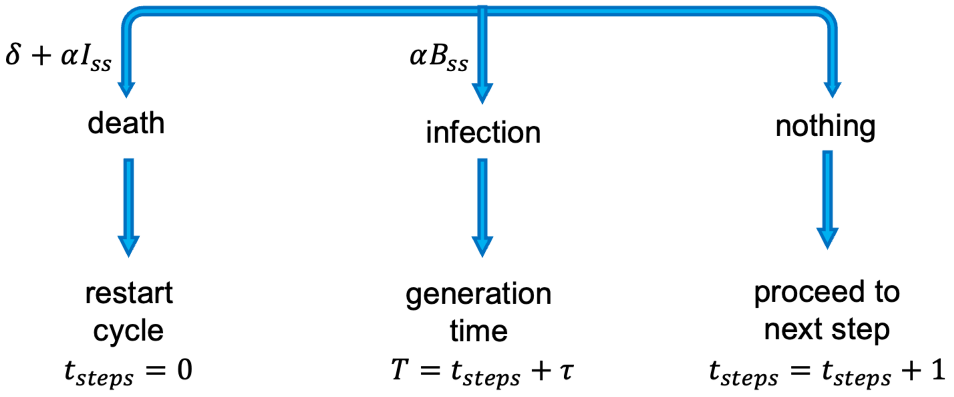

Figure E1: Schematic diagram illustrating the processes used to verify the generation time T in superinfection-excluding populations.

Throughout the main text we use the above generation time for both superinfection-excluding and superinfecting populations. However, the superinfection scenario differs from that laid out above in that adsorption to infected cells does not result in death, and relatedly, the time between successful host infection and offspring production may be less than τ. To evaluate the error we introduce with our approximation of generation time, we modify the simple stochastic simulations above to take into account these differences. In this scenario, in a single time-step the phage has a probability $\delta \Delta t$ of dying, and a probability $\alpha B_0 \Delta t$ of infecting a host (either infected or uninfected). In the case where infection occurs, the phage has a probability B_{ss}/B_0 of infecting an uninfected host, which as before, results in a generation time $T = \tau + t_{steps}$. In the remainder of infection cases, phage will infect already infected hosts. Because of the nature of the process of within-host replication, secondary infections that occur too late after the initial infection generate almost no offspring of the superinfecting phage. We account for this observation by assuming that only secondary infections occurring within the first n steps post initial infection will successfully produce offspring. Given that we are considering populations at steady state, we assume that infected cells are equally likely to be found any number of steps post-infection ($< \tau$), and so infection a cell in the first n steps post initial infection simply occurs a fraction n/τ of the times that secondary infection occurs. In this case, the generation time is given by $T = t_{steps} + \tau - \frac{\sum_1^n k}{n}$, where the

final two terms represent the average number of steps between secondary infection and lysis. This final term can be simplified by noting that $\frac{\sum_1^n k}{n} = \frac{n(n+1)}{2n} = \frac{n+1}{2}$. A schematic representation of this process is shown in Fig E2.

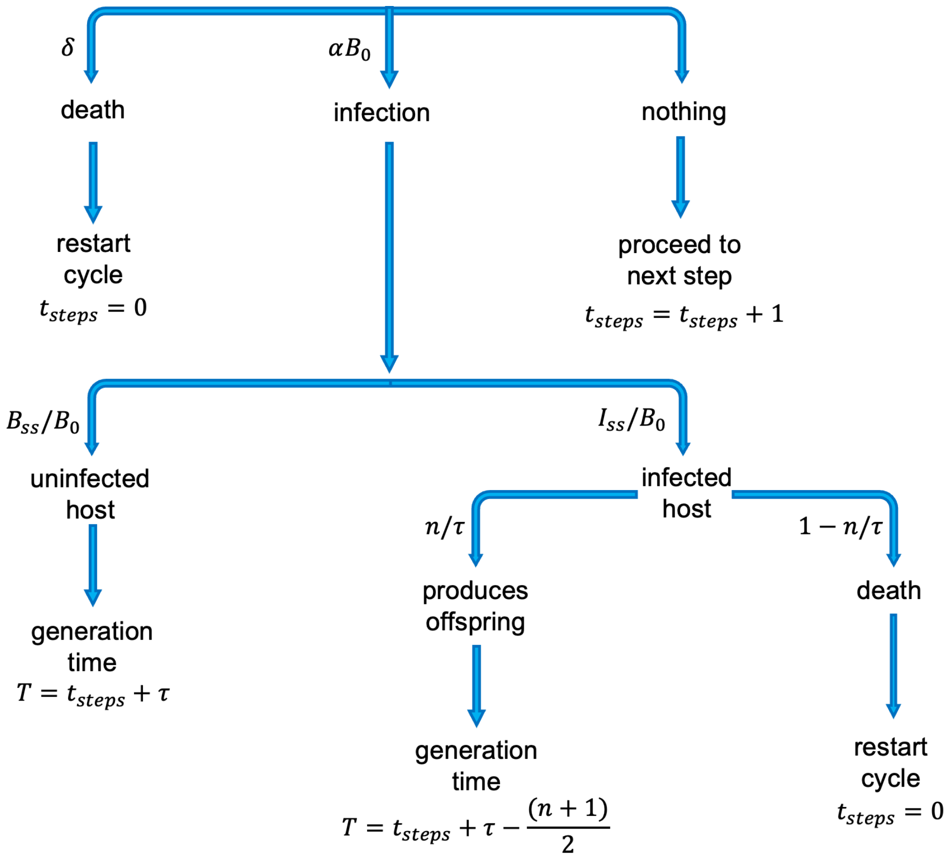

Figure E2: Schematic diagram illustrating the processes used to measure the generation time T in superinfecting populations.

Using a value of $n = 3$, this process was again repeated 10 million times, yielding an average generation time of $\overline{T} = 24.11(3)$. It can be seen that the difference in generation time is marginal. It is however worth noting that were we to fully account for the shorter generation time in superinfecting populations, it would result in an even larger effective population size, further emphasising our main findings.
