# Supplementary material for: Superinfection exclusion: A viral strategy with short-term benefits and long-term drawbacks
Source: PLoS Comput Biol. 2022 May 10;18(5):e1010125. doi: 10.1371/journal.pcbi.1010125 (PMC9122224; doi:10.1371/journal.pcbi.1010125)
Supplement: Attachment — Submitted filename: Comments to the authors.pdf [file pcbi.1010125.s010.pdf]

In this work, Hunter and Fusco explore some evolutionary consequences of superinfection exclusion using computational simulations that describe the dynamics of a phage population in a turbidostat system. This system maintains a constant number of cells analogously to the Moran model typically used to describe the evolution of finite populations of haploid replicators. They used simulated data to estimate key population genetics parameters such as the effective population size, fixation probabilities and selection coefficients to disentangle the course of neutral, advantageous and detrimental mutations. Furthermore, they have observed that, by limiting the competitive ability of superinfecting viruses, SIE mechanisms provide a short-term selective benefit to the virus even if these incur substantial costs on viral growth rate, making SIE a winning strategy even though promoting superinfection may lead to long-term positive effects on viral adaptation.

SIE is a long-known and mechanistically diverse phenomenon that has been observed in multiple animal, plant and bacterial viruses. However, despite this ubiquity, its evolutionary meaning and consequences have rarely been considered. During recent years, applying social evolution to viruses has increased awareness of the importance of virus-virus interactions (such as SIE) for viral dynamics, ecology and evolution. Therefore, I find this work valuable as a first theoretical approximation to describe the impact of SIE on viral evolutionary dynamics, and I believe that it has the potential to promote other, more detailed theoretical and experimental analyses. However, I am concerned about the validity of some results because of a particular assumption of the computational framework (see below). I also have other comments that I hope may help the authors to revise and improve their work.

MAIN CONCERN

(I) The authors simulate a well-mixed population of phages replicating in a population of bacteria kept at a constant population size (turbidostat system). Phage growth is determined by a set of life history parameters, which are the adsorption rate α, the lysis time τ, and the burst size β. In both superinfecting and superinfection-excluding scenarios, when two different phages infect the same cell, it is assumed that β and τ are established by the first virus to infect (lines 116-118, 127-129). This means that both viruses replicate at the same constant pace β/τ.

To me, this assumption seems non-informative and unrealistic because selection cannot favor a mutant over the other if both replicate at the same rate and produce the same burst size. This may have important consequences at least for the results obtained for different values of β and τ parameters. For example, the conclusion that "Higher growth rate does not translate into competitive advantage" is probably conditioned by the aforementioned assumption. Authors found that changes in the adsorption rate α causes larger effects on selection coefficients (both in isolation and under competition) than changes in β, whereas lysis time τ produces no effect in a competition setting (which contradicts previous works and intuition as authors indicated in lines 216-220 and 322-325). In lines 220-223 and 325-334, it is suggested that the lack of effect of τ mutants on the outcome of competitions is due to the mutants being introduced into the turbidostat system once equilibrium is reached. To show that, they used a system of delay differential equations, which properly describes simulated dynamics (Appendix S1). However, it should be noted that in this deterministic model, as long as you don't get a cyclic steady state, τ loses its meaning at the equilibrium, since $V_t = V_{t-\tau}$ and $B_t = B_{t-\tau}$.

The way I see it, the effects of altering life history parameters presented in Figs. 4 and S2 can be explained as follows:

- In a competitive setting, changes in α affect the number of adsorption events in a time step, which determines the differential contribution of the α mutant to the infection (and its probability of being first to infect).

- Changes in τ have no effect on competition since both phages (WT and mutant) display the same (per phage) probability of being first (same α) and the same burst size (same β). Therefore, as both replicate at the same rate, the contribution of both phages to the final progeny is equal on average.

- Even if both phages replicate at the same rate in coinfection, changes in β cause differential growth in competition because the phage displaying higher burst size gains a competitive advantage when it is the one initiating cell infection, since in these instances it tends to receive a greater share of progeny (p_x) at the lysis time.

I think that it would be much more realistic (assuming independent replication) to consider that phage_a replicates at rate β_a/τ_a and phage_b does so at a rate β_b/τ_b. Then, the first phage to reach its burst would induce cell lysis, and the replicated progeny would be released accordingly to the number of viruses produced applying a process similar to that described in lines 134-138.

OTHER COMMENTS

(II) I would not say that MOI > 1 is a necessary condition for superinfection, as authors indicated in lines 52-54. High MOI is necessary for coinfection in the absence of collective dispersal mechanisms. However, superinfection does not involve the simultaneous infection of uninfected cells by several viruses, but the secondary infection of a previously infected cell. Therefore, superinfection may occur at low MOI in many cases where primary infections are abundant. For example, consider a colony of lysogenic bacteria. The probability that a particular superinfectant phage will infect a lysogen will depend on the frequency of lysogens in the colony, being completely independent of the MOI if this frequency is 100%. I would also be cautious on using superinfection and coinfection as synonyms, as the authors seems to do sometimes (lines 52-54, 62-65, 67-69, 192, 309). Although many results obtained in other works analyzing the evolutionary implications of coinfection or collective dynamics could probably be applied to superinfection (at least qualitatively), others may not be adequate. For this reason, if possible, it might be better to indicate when the cited literature refers to results obtained from coinfection or superinfection settings.

(III)

From a social evolutionary perspective, social traits are those that evolve not only by their effects on the individuals who carry them, but also by their effects on other

individuals. There is no clear trait behind superinfection, and it cannot be considered as a cooperative or social strategy (as it is proposed in line 339), because it simply promotes competition for finite host resources. Moreover, the phage sharing these resources does not receive any positive effect on its fitness due to the presence of the other phage.

The fitness of individuals is represented by their reproductive capacity under particular circumstances, not by the adaptive capacity of the population (as indicated in lines 340-348 and also 12-13). Adaptive capacity refers to how it is expected that the fitness of individuals in a population evolve over time under different selective pressures. Therefore, interpreting that phages allowing superinfection are “fitter” because they can be expected to be better adapted and exhibit greater long-term fitness is a qualitative statement that does not seem appropriate to formulate a payoff matrix that should contain the fitness of interacting individuals according to the strategies they “play”.

What is discussed in lines 335-351 is not analogous to the case of Turner and Chao 1999 (as indicated in lines 335-337). In that instance, coinfecting viruses cooperate because they pay a cost to produce gene products that are shared as a public goods. These public goods may be exploited by other cheating viral variants (defectors) who gain a benefit without reciprocating. In that case, the payoff matrix was constructed with the fitness of individuals depending on whether they cooperate or defect with other cooperators or defectors. A cooperator-only population had higher fitness (but this is not in the long run). However, because payoffs in the Prisoner’s dilemma game are arranged as $W_{D|C} > W_{C|C} > W_{D|D} > W_{C|D}$, natural selection promoted the evolution of defectors (which is correctly explained in lines 348-351). If needed, some articles may help to clarify this ideas: West 2007 (PMID: 17305808), Nowak 2006 (PMID: 17158317), Díaz-muñoz 2017 (PMID: 29024640), and Sanjuan 2021 (PMID: 34242062).
