# Supplementary material for: Superinfection exclusion: A viral strategy with short-term benefits and long-term drawbacks
Source: PLoS Comput Biol. 2022 May 10;18(5):e1010125. doi: 10.1371/journal.pcbi.1010125 (PMC9122224; doi:10.1371/journal.pcbi.1010125)
Supplement: Attachment — Submitted filename: Reply_PLOS_mth47.docx [file pcbi.1010125.s011.docx]

Dear Dr. Illingworth & Dr. Mustonen,

Thank you for your consideration of our manuscript entitled *Superinfection exclusion: a viral strategy with short-term benefits and long-term drawbacks,* and for giving us the opportunity to submit a revised version that addresses the reviewers’ comments.

We are glad that Reviewer #1 enjoyed reading our manuscript, and that they believed it to be of value to the field. In their report, the reviewer had several minor comments related to areas where they felt like additional exposition would aid the reader’s understanding. As a result, we have made several changes to the text in an attempt to clarify the various aspects which the reviewer highlighted (details included in following point-by-point response). We have also included an additional Appendix to the manuscript, where the decision not to introduce stochasticity in lysis time is discussed and explored with additional simulations. We believe that these changes will improve the reader’s understanding of our main points, and clarify the reasoning behind some aspects of our model.

We are also glad that Reviewer #2 found our work to be of value as a first theoretical exploration that can promote further analyses in the field. In their report, the reviewer expressed concerns about our computational framework, specifically the decision to set a constant growth rate of phage inside the host cell after superinfection, which is determined by the first phage to infect. The reviewer points out that this decision limits the ability for selection to favour one mutant over another, as both grow at the same rate inside the cell. We completely agree with this point, and, as a result, we have changed our computational framework so that the phage inside the host grow at a rate determined by their own parameters, rather than the parameters of the first phage to infect. We have re-run the relevant simulations, and these results are included in the revised manuscript. In most instances, this does not qualitatively alter our findings, and so does not alter the conclusions of our work. The most significant change relates to alterations in lysis time, which in the superinfecting regime now lead to a detectable (dis)advantage in a competitive setting, although still at a significantly reduced level when compared to the impact on growth rate. Importantly, this finding increases the emphasis of our previous results, as selection in this instance is now *much* more efficient in the superinfecting regime than in the superinfection-excluding one. The reviewer also had some other more minor comments relating mainly to terminology and analogies drawn in our work, which we have attempted to address.

We hope that these changes will make the manuscript suitable for publication in *PLoS Computational Biology.* As requested, please find below our point-by-point response to all of the reviewers’ comments and criticisms, alongside the revised manuscript for your consideration.

Yours sincerely,

Michael Hunter & Diana Fusco

Cavendish Laboratory

University of Cambridge

Cambridge, CB3 0HE, UK

mth47@cam.ac.uk

Response to Reviewer #1

*Reviewer #1: Overall, I really enjoyed this paper. The question of whether superinfection exclusion poses advantages or disadvantages to the evolution of phage populations is a longstanding one, but the authors manage to present their approach in a way that appears straightforward while also making a nice contribution to the field. I find that their results justify their conclusions, though I have several minor points that I think could be addressed to make the work even more clear. There were a few areas where I think additional exposition would help the reader understand the authors’ points.*

We thank the reviewer for their support of our work, and welcome the opportunity to clarify the aspects suggested by the reviewer below.

*I found the introduction well-written and it poses the questions the authors aim to address very clearly. I think that the breadth of literature covered in the introduction does a good job of summarizing the gaps of knowledge in this sub-field and where progress has been made so far. I would also recommend citing the sociovirology review written by Sam Diaz-Munoz, Rafael Sanjuan, and Stuart West (10.1016/j.chom.2017.09.012), which is in my view one of the more important pieces written on the topic.*

We have followed the reviewer’s suggestion and added this citation to the introduction.

*Lines 85-86: The authors state that adsorption that happens at times later than the first adsorption event do not contribute DNA to the host cell in the superinfection excluding scenarios. Does this mean that the phages that enter later are removed from the free phage population, but do not get replicated? This would be in contrast, I think, to what is described in the Superinfecting section that begins on line 126 where subsequent adsorption events increase the within-cell population until lysis. Lines 123 to 125 may be a good place to clarify.*

We choose to model a type of superinfection exclusion mechanism which does not prevent subsequent adsorption events (as in T5), but does prevent the injection of subsequent DNA, as it occurs in T3, T4 and T7. As the reviewer says, this means that in the superinfection excluding scenario phages which attempt infection of a previously infected cell do adsorb to the host, so are removed from the free phage population, but do not get replicated. The key difference between the superinfecting and superinfection-excluding scenarios is that in the former secondary infecting phage can replicate within the host, while in the latter they do not contribute to the within-cell population and effectively die.

*Lines 121-123: The lysis time is not drawn from a distribution with parameter tau, unlike burst size which has mean beta, or the number of phage that adsorb in a timestep which depends on alpha. Why lysis time would not be stochastic, but burst size and adsorption are stochastic, is not clear to me so I would appreciate it if the authors motivated this choice.*

While adsorption rate and burst size can be modelled stochastically without the need of introducing additional parameters by using a Poisson distribution, modelling stochasticity in lysis time in a realistic fashion would require additional parameters. For instance, Campos *et al.* 2008 (10.1016/J.JTBI.2008.05.035) compare descriptions of lysis using piece-wise, Gaussian and logistic-like functions. Depending on the mathematical framing, each of these implementations would require at least one parameter that controls the width of the distribution. Given that our model already contains many parameters, we thought it better to choose a simpler description, where lysis time is deterministic.

We appreciate, however, the reviewer comment and implemented a version of our model where lysis time is drawn stochastically from a Gaussian distribution with a set standard deviation and re-run a subset of our simulations to demonstrate that the results are not significantly altered by the inclusion of a stochastic lysis time. These results, and a discussion of our motivation for not including lysis stochasticity in the main model are now included as Appendix S1.

*Lines 194-197: The juxtaposition here is throwing me. Why would a neutral mutant have two cell types available to it while the wild-type can only infect uninfected cells? Is it because we are at steady state when the mutant infects, so it has multiple cell-states available, whereas the wild-type before steady state only expanded through uninfected cells? Please elaborate.*

Crucially, at the moment in which the mutant is introduced to the free phage population, all of the cells that are already infected are infected by wild-type phage. In the superinfecting scenario, the mutant population can therefore grow by infecting an uninfected cell, or by infecting an already infected cell, as this secondary infection will lead to some fraction of the burst size being allocated to the mutant type. While wild-type phage can also replicate by infecting infected cells, the wild-type population won’t be affected by these events, because all infected cells are already exclusively infected with wild-type phage. As a result, superinfection of wild-type infected cells by more wild-type phage does not result in any more wild-type phage being produced. We have modified the text in an attempt to clarify this.

*Line 206: You introduce parameter s here, but I don’t think it was defined in the methods. Is it just the general term for s_growth and s_comp? If so, please state.*

Its use here was indeed meant to simply represent a general term for *s_growth* and *s_comp*. To avoid any confusion, we have simply removed the parameter *s* from this sentence as it is not needed.

*Line 252: How do sigma and phi relate to each other? You describe them as fitting and scaling parameters for the various s variables, but their roles are unclear.*

Sigma is a parameter that relates the fitness in a competitive setting to the fitness in terms of growth rate (i.e. s_comp=sigma*s_growth). In the revised manuscript, it is now explicitly stated that this parameter is obtained from linear fits to the data presented in Fig. 4 in all cases. We have made changes to the text in an attempt to clarify this.

Phi is a parameter we use to quantitatively evaluate how well our data maps to the prediction made by an equivalently parameterised Moran model. In the revised manuscript, we introduce phi as s_comp=phi*s_growth, as with sigma, but where phi is determined by fitting the fixation data presented in Fig. 5 to the Moran model. By comparing our values for phi and sigma in each case we can then quantitatively assess the degree to which our model can be described by an equivalently parameterised Moran process.

*Figure 6: It would be nice if you listed the number of simulations ran for each of your figures and their subplots. It is in the methods for some of the work but I don’t think it encompasses all of the figures. looks like 1/10^6.5 based on 6b if the leftmost point has been adjusted given your absence of observed fixation. I do appreciate that they state the limitation that they did not observe fixation here, though would the value plotted be an upper bound instead of a lower bound like the authors write? More simulations to observe a rare fixation event would decrease the estimated probability, no?*

The minimum number of simulations ran for the data in each of the figures has now been added to the figure captions.

In Fig. 6 (and in the other figures) we have not “adjusted” any data points due to an absence of observed fixation – if no fixation was observed, there is no data point. Data points only represent cases where fixation was observed.

With regards to the lower/upper bound: in Fig. 6 we run a minimum of 20 million simulations, meaning that we should have the power to detect a probability of fixation of ~10^-7. The fact that we see no fixation events therefore implies that the probability of fixation is <10^-7. Given that the initial frequency of the mutants is ~10^-5, we therefore say that in the case where no fixation is observed, the mutants fix at least 2 orders of magnitude less frequently than would be expect for a neutral mutant. It is this value of “2” orders of magnitude which we had referred to as a lower bound, because in reality the fixation events are likely even more rare than this (as the reviewer says), and so more than 2 orders of magnitude less likely to fix. We have made changes to the text in an attempt to clarify.

*Figure 6: The error bars need to be clarified. I assume it has something to do with s_growth reduction but it really isn’t clear to me how the bars relate to the points as they seem to be varying different parameters, but wouldn’t an additional dimension need to be displayed to adequately show the exploration of this parameter space?*

The error bars in the y-axis in this figure are given by the number of fixation events we observe assuming a Poisson distribution, i.e. 1/sqrt(n_fix). In the x-axis, the error bars are based on our growth rate measurements. By measuring the growth rate, over the range of beta explored here we are able to find a linear relation between the burst size of a mutant and the growth rate fitness i.e. s_growth=(beta_mutant-beta_wt)*gradient. Our error in growth rate is then determined from the error on our best fit gradient, with the fractional error on s_growth being the same as the fractional error on the gradient. We have added this information to the figure caption (and the captions of the other figures) to clarify.

*Lines 328-330: I think this is an excellent point.*

We are glad the reviewer approves of this point.

*Line 404: Is this alpha value consistent with what is explored in the figures? Here you state that the empirical value is on the order of 10^-9, but the parameters explored in the figures are on the order of 10^-6.*

Our values are chosen such that the product of alpha*B0 is consistent with empirical values from classic adsorption measurements performed in cultures with typically 10^7 or 10^8 cells per ml (3*10^-3 per min in our simulations vs 10^-3 – 10^-1 per min experimentally). This is so that the adsorption timescales remain consistent with the lysis timescales. We use larger adsorption rates and smaller bacteria population sizes for computational reasons i.e. decreasing the adsorption rate by a factor of 10^3, and increasing the bacteria density by a factor of 10^3 would yield simulations that are too computationally intensive to yield results in sensible timescales. We have added a sentence to the methods to clarify this decision.

*I thought that the Methods section was very well written – nice work. Particularly the section on calculating the fixation probability.*

We are glad the reviewer thought the Methods section was well written, and offer our thanks.

*I also found several spelling errors, so I think the authors should give a careful read if they resubmit. Examples: Figure 1 caption: “superinfecting in permitted” should instead be “is permitted” Line 332: “our results suggests” should be “suggest”*

We thank the reviewer for highlighting these errors, they have been addressed.

Response to Reviewer #2

*In this work, Hunter and Fusco explore some evolutionary consequences of superinfection exclusion using computational simulations that describe the dynamics of a phage population in a turbidostat system. This system maintains a constant number of cells analogously to the Moran model typically used to describe the evolution of finite populations of haploid replicators. They used simulated data to estimate key population genetics parameters such as the effective population size, fixation probabilities and selection coefficients to disentangle the course of neutral, advantageous and detrimental mutations. Furthermore, they have observed that, by limiting the competitive ability of superinfecting viruses, SIE mechanisms provide a short-term selective benefit to the virus even if these incur substantial costs on viral growth rate, making SIE a winning strategy even though promoting superinfection may lead to long-term positive effects on viral adaptation.*

*SIE is a long-known and mechanistically diverse phenomenon that has been observed in multiple animal, plant and bacterial viruses. However, despite this ubiquity, its evolutionary meaning and consequences have rarely been considered. During recent years, applying social evolution to viruses has increased awareness of the importance of virus-virus interactions (such as SIE) for viral dynamics, ecology and evolution. Therefore, I find this work valuable as a first theoretical approximation to describe the impact of SIE on viral evolutionary dynamics, and I believe that it has the potential to promote other, more detailed theoretical and experimental analyses.*

We thank the reviewer for their support of our work and its value in promoting further analyses.

*However, I am concerned about the validity of some results because of a particular assumption of the computational framework (see below). I also have other comments that I hope may help the authors to revise and improve their work.*

*MAIN CONCERN*

*The authors simulate a well-mixed population of phages replicating in a population of bacteria kept at a constant population size (turbidostat system). Phage growth is determined by a set of life history parameters, which are the adsorption rate α, the lysis time τ, and the burst size β. In both superinfecting and superinfection-excluding scenarios, when two different phages infect the same cell, it is assumed that β and τ are established by the first virus to infect (lines 116-118, 127-129). This means that both viruses replicate at the same constant pace β/τ.*

*To me, this assumption seems non-informative and unrealistic because selection cannot favor a mutant over the other if both replicate at the same rate and produce the same burst size. This may have important consequences at least for the results obtained for different values of β and τ parameters. For example, the conclusion that “Higher growth rate does not translate into competitive advantage” is probably conditioned by the aforementioned assumption. Authors found that changes in the adsorption rate α causes larger effects on selection coefficients (both in isolation and under competition) than changes in β, whereas lysis time τ produces no effect in a competition setting (which contradicts previous works and intuition as authors indicated in lines 216-220 and 322-325). In lines 220-223 and 325-334, it is suggested that the lack of effect of τ mutants on the outcome of competitions is due to the mutants being introduced into the turbidostat system once equilibrium is reached. To show that, they used a system of delay differential equations, which properly describes simulated dynamics (Appendix S1). However, it should be noted that in this deterministic model, as long as you don’t get a cyclic steady state, τ loses its meaning at the equilibrium, since Vt= Vt-τ and Bt=Bt-τ.*

*The way I see it, the effects of altering life history parameters presented in Figs. 4 and S2 can be explained as follows:*

*- In a competitive setting, changes in α affect the number of adsorption events in a time step, which determines the differential contribution of the α _mutant to the infection (and its probability of being first to infect).*

*- Changes in τ have no effect on competition since both phages (WT and mutant) display the same (per phage) probability of being first (same α) and the same burst size (same β). Therefore, as both replicate at the same rate, the contribution of both phages to the final progeny is equal on average.*

*- Even if both phages replicate at the same rate in coinfection, changes in β cause differential growth in competition because the phage displaying higher burst size gains a competitive advantage when it is the one initiating cell infection, since in these instances it tends to receive a greater share of progeny (px) at the lysis time.*

*I think that it would be much more realistic (assuming independent replication) to consider that phage_a replicates at rate βa/τa and phage_b does so at a rate βb/τb. Then, the first phage to reach its burst would induce cell lysis, and the replicated progeny would be released accordingly to the number of viruses produced applying a process similar to that described in lines 134-138.*

We thank the reviewer for the detailed suggestion on the model, which we take fully in consideration in the following. As we noted in the original manuscript, the decision to set the burst size, lysis time and internal growth rate according to the first phage to infect was made for the sake of simplicity, given the wide range of superinfecting behaviours observed. We do agree, however, with the reviewer’s main concern that this choice limits the ability for selection to favour one mutant over another as it effectively means that competition only occurs outside the cell, rather than also inside the cell in the superinfecting scenario.

To address this, we have changed our model in the following 2 ways:

1) Inside the cell, each phage now grows at a rate determined by its own parameters, rather than the parameters of the first phage to infect. As before, however, the actual amount of growth achieved by each phage pseudo-population inside the host depends on the relative size of both populations at each point in time, i.e. p_a increases by an amount (beta_a/tau_a)*(p_a/(p_a+p_b)) in a given timestep while p_b increases by an amount (beta_b/tau_b)*(p_b/(p_a+p_b)). We think that this maintains the sense that there is a fixed amount of resources (e.g., ribosomes) available to the phages inside the host, while allowing for mutants to use these resources more/less efficiently by having an altered burst size or lysis time.
2) To determine the exact number of phages released upon lysis, we draw a random number from a Poisson distribution with mean equal to the sum of the two pseudo-populations at the time of lysis (minus however many phages had infected the host up until this point), rather than a mean equal to the burst size of the first infecting phage. This means that if a cell is only infected by 1 type of phage, its mean burst size remains beta, regardless of how many other identical phages had infected the cell until that point.

We believe that these changes address the principal concern of the reviewer, as selection now has the opportunity to act while the phage are inside the host, since alterations to the life-history parameters will affect both the total burst size of the cell, and the way in which that burst size is partitioned to each type of phage.

In the new version of the model, we do however keep the decision that the eventual lysis time is set by the first phage to infect. The reason for this is primarily simplicity, as the effect of multiple infections is not consistent across different species/interactions. For instance, multiple infections can result in lysis at the expected time of the original infector, but with a significantly reduced burst size, while in other scenarios lysis can be delayed, with the total burst size released being larger than expected for a single infection (see, for instance, M. Adams *Bacteriophages* 1959). The second reason is due to the fact that in the scenarios we consider, differences in lysis time are small. If we imagine that each phage produces some “lysis products” once inside the host at a rate 1/tau, and lysis is triggered once the total amount of lysis product reaches 1, due to the partitioning of resources previously described, and the very small differences in lysis time that we look at between mutant and wild type, the difference that we would introduce to the lysis time is less than the size of a timestep that we look at in our simulations.

The text has been re-written to reflect these new modelling choices, and the figures have been updated with the new results. In the case of altered burst size, the difference between superinfecting and superinfection-excluding scenarios is now slightly more significant, i.e. selection is even more efficient in the superinfecting case than it was in the previous version of the model.

In the superinfecting scenario, differences in lysis time now lead to an observable fitness (dis)advantage in a competitive setting, although this impact remains much less than the impact on growth rate (i.e. s_growth>s_comp). This impact is also reflected in the probability of fixation of single mutants, with the behaviour still showing good agreement with an equivalently parameterised Moran model. Our intuition for this result remains the same: we believe that this is a special feature of a turbidostat setting, as lysed hosts are immediately replaced by uninfected cells, providing the same number of viable hosts independently of the time needed by the phage to lyse them. As the reviewer notes, once at equilibrium, tau loses its meaning in the deterministic ODE model, which captures the average behaviour of the model, at least in the superinfection-excluding case. In this new version of the model however, we do observe a selective (dis)advantage in direct competition in the superinfecting scenario. We believe that this effect arises because, while the extracellular competition is limited by the turbidostat setup, now in the superinfecting scenario there is the opportunity for some intracellular competition to occur, as mutants will grow at different rates inside the host. Importantly, this new result emphasizes even more how superinfection is expected to be evolutionary advantageous to fix beneficial mutations and purge deleterious once, corroborating our main point.

*OTHER COMMENTS*

*(II) I would not say that MOI > 1 is a necessary condition for superinfection, as authors indicated in lines 52-54. High MOI is necessary for coinfection in the absence of collective dispersal mechanisms. However, superinfection does not involve the simultaneous infection of uninfected cells by several viruses, but the secondary infection of a previously infected cell. Therefore, superinfection may occur at low MOI in many cases where primary infections are abundant. For example, consider a colony of lysogenic bacteria. The probability that a particular superinfectant phage will infect a lysogen will depend on the frequency of lysogens in the colony, being completely independent of the MOI if this frequency is 100%. I would also be cautious on using superinfection and coinfection as synonyms, as the authors seems to do sometimes (lines 52-54, 62-65, 67-69, 192, 309). Although many results obtained in other works analyzing the evolutionary implications of coinfection or collective dynamics could probably be applied to superinfection (at least qualitatively), others may not be adequate. For this reason, if possible, it might be better to indicate when the cited literature refers to results obtained from coinfection or superinfection settings.*

In part our somewhat synonymous use of the terms co-infection and superinfection is due to their often synonymous and inconsistent usage in phage literature – see Abedon 2015 (10.1007/s12250-014-3547-2) for a discussion of the ambiguity and inconsistency in the use of terms secondary adsorption, secondary infections, superinfection and coinfection in phage literature. Turner & Duffy 2008 (10.1017/CBO9780511541483.011) define the terminology such that “Two phages which have infected a single bacterial cytoplasm can be said to have coinfected,” and that “superinfection is a consequence of a delay in infection by a second phage of an already infected bacterium.” Therefore, as they state, “all bacteria that have been successfully superinfected can be said to have been coinfected.”

By this definition, all of the phage literature which refers to “superinfection” is also referring to coinfection. Similarly, much of the work which refers to “co-infection” is likely also at least in part referring to superinfection. Many of the computational papers in this area for instance which look at “co-infection” explicitly incorporate delayed secondary infections in their model, or consider only within-host dynamics without describing the actual infection dynamics at all. Similarly in experimental papers, co-infection likely occurs due to both simultaneous and delayed secondary infections in many cases.

In an attempt to address this ambiguity, we have added a short section at the start of the introduction where we clarify our usage of the terminology in line with the definitions given by Turner & Duffy above. When discussing the cited literature in our introduction, we now also refer to “multiple infections” rather than either superinfection or co-infection. This term has also been used throughout much of the cited literature, and is more broadly applicable when discussing viruses beyond phage.

When discussing our own work however, we continue to use the term superinfection. This is because, while in principle in our simulations multiple phages can infect a single host in a given timestep, this is due to the discretised nature of time in our simulations. In the event that this occurs, we still choose one of the phages to be the first, or primary, infector. There are therefore no simultaneous primary infections, and so all multiple infections in our work arise from superinfection.

We also take the reviewer’s point about MOI>1 not being “necessary” in all situations. We have therefore removed this statement, and replaced it with a statement that multiple infections become more likely as MOI increases.

*(III) From a social evolutionary perspective, social traits are those that evolve not only by their effects on the individuals who carry them, but also by their effects on other individuals. There is no clear trait behind superinfection, and it cannot be considered as a cooperative or social strategy (as it is proposed in line 339), because it simply promotes competition for finite host resources. Moreover, the phage sharing these resources does not receive any positive effect on its fitness due to the presence of the other phage.*

*The fitness of individuals is represented by their reproductive capacity under particular circumstances, not by the adaptive capacity of the population (as indicated in lines 340-348 and also 12-13). Adaptive capacity refers to how it is expected that the fitness of individuals in a population evolve over time under different selective pressures. Therefore, interpreting that phages allowing superinfection are “fitter” because they can be expected to be better adapted and exhibit greater long-term fitness is a qualitative statement that does not seem appropriate to formulate a payoff matrix that should contain the fitness of interacting individuals according to the strategies they “play”.*

*What is discussed in lines 335-351 is not analogous to the case of Turner and Chao 1999 (as indicated in lines 335-337). In that instance, coinfecting viruses cooperate because they pay a cost to produce gene products that are shared as a public goods. These public goods may be exploited by other cheating viral variants (defectors) who gain a benefit without reciprocating. In that case, the payoff matrix was constructed with the fitness of individuals depending on whether they cooperate or defect with other cooperators or defectors. A cooperator-only population had higher fitness (but this is not in the long run). However, because payoffs in the Prisoner’s dilemma game are arranged as WD|C > WC|C > WD|D > WC|D, natural selection promoted the evolution of defectors (which is correctly explained in lines 348-351). If needed, some articles may help to clarify this ideas: West 2007 (PMID: 17305808), Nowak 2006 (PMID: 17158317), Díaz-muñoz 2017 (PMID: 29024640), and Sanjuan 2021 (PMID: 34242062).*

Our motivation for drawing this comparison to the Prisoner’s Dilemma was simply intended as a parallel perspective that readers in the field may find interesting. However, we do appreciate the reviewer’s points that our situation cannot strictly be formulated in this fashion, as we are conflating long-term population benefits (i.e. increased selection efficiency and adaptability) with short-term individual benefits (i.e. a mutant’s probability of fixing). We have therefore simply removed this paragraph from our discussion, as it is not necessary for our conclusions.

*Have the authors made all data and (if applicable) computational code underlying the findings in their manuscript fully available?*

*Reviewer #2:* ***No:*** *The authors provide a link to their code in the manuscript, but for some reason it cannot be accessed.*

Our apologies, this was a mistake on our part. The repository was private rather than public, and so was not accessible. This issue has now been fixed, and the repository has been updated to reflect the new code used in the revised manuscript.
